# Effects of Thoracentesis in Patients Under Invasive Mechanical Ventilation: A Retrospective Analysis of Clinical and Paraclinical Parameters

**DOI:** 10.3390/jcm15083133

**Published:** 2026-04-20

**Authors:** Danilo Andrés Cáceres-Gutiérrez, Héctor Fabio Escobar-Vargas, Diana Marcela Bonilla-Bonilla, Jorge Enrique Daza-Arana, Heiler Lozada-Ramos, María Angelica Rodríguez-Scarpetta

**Affiliations:** 1 Specialization in Internal Medicine, Faculty of Health, Universidad Santiago de Cali, Cali 760035, Colombia; danilo.caceres00@usc.edu.co (D.A.C.-G.); diana.bonilla01@usc.edu.co (D.M.B.-B.); jorge.daza01@usc.edu.co (J.E.D.-A.); heiler.lozada00@usc.edu.co (H.L.-R.); 2 Departament of Research and Education Department, Clínica de Occidente S.A., Cali 760035, Colombia; hector.escobar00@usc.edu.co; 3 Physiotherapy Program, Faculty of Health, Universidad Santiago de Cali, Cali 760035, Colombia

**Keywords:** thoracentesis, pleural effusion, exudate, transudate, mechanical ventilation, left ventricular ejection fraction

## Abstract

**Background**: Thoracentesis is pivotal in managing pleural effusion (PE), particularly in invasive mechanical ventilation (IMV), with documented improvements in respiratory mechanics, oxygenation, and hemodynamic parameters. However, its efficacy may vary based on effusion type and drained volume. **Methods**: A retrospective longitudinal study was conducted at a high-complexity care center in Cali, Colombia (2019–2024), including 93 (IMV) patients who underwent therapeutic thoracentesis (TT). Respiratory and hemodynamic parameters were assessed before and up to 24 h post-procedure. Stratified analysis was performed by drained volume, fluid type, and left ventricular ejection fraction (LVEF). **Results**: TT yielded significant improvements in fraction of inspired oxygen (FiO_2_) (−4%), positive end expiratory pressure (PEEP) (−0.5 cmH_2_O), and Oxygen arterial Pressure Index/Inspired Oxygen Fraction (PaO_2_/FiO_2_-ratio) (+27.1), with greater impact for volumes ≥500 mL and transudative PE. Patients with LVEF ≤ 40% showed increased mean arterial pressure (MAP) and PaO_2_. Complication rates were low (<4%). **Conclusions**: TT is safe and effective in critically ill IMV patients, particularly for transudative PE and drained volumes ≥500 mL, as well as in subjects with LVEF ≤ 40%. Its positive impact on oxygenation and ventilation supports its therapeutic utility in critical care.

## 1. Introduction

Thoracentesis is a low-risk, effective procedure; however, complications such as pneumothorax, hemorrhage, or re-expansion pulmonary edema may increase morbidity, mortality, and hospital costs. For instance, iatrogenic pneumothorax can prolong hospitalization by 4.4 days [1]. Notably, complication rates vary by technique [2].

Approximately 41% of ICU patients develop PE [3,4], potentially compromising hemodynamics, respiratory mechanics, and gas exchange. TT may improve pulmonary function by facilitating re-expansion of collapsed parenchyma, enhancing ventilatory mechanics, ventilation perfusion matching, and oxygenation. A critical step in pleural fluid evaluation is distinguishing exudative (inflammatory) from transudative (non-inflammatory) PE. Since its introduction in 1972, Light’s criteria remain the diagnostic standard: PE is classified as exudative if ≥1 of the following is met: pleural/serum protein ratio > 0.5, pleural/serum lactate dehydrogenase (L) ratio > 0.6, or pleural LDH > 200 IU/L (>67% of serum LDH upper limit) [5].

Prior studies evaluating TT’s effects on hemodynamic and respiratory function in ICU patients under IMV have measured parameters such as PaO_2_/FiO_2_, alveolar-arterial (A-a) gradient, end-expiratory lung volume, heart rate (HR), and LVEF, alongside complications like hemothorax and pneumothorax. Several studies report that pleural drainage improves PaO_2_/FiO_2_, dynamic compliance, and PaO_2_ while reducing respiratory work and rate [6,7,8,9]. These benefits appear more pronounced with larger drained volumes (>500 mL) [3,10,11]. Likewise, when small volumes are evacuated, complications such as pneumothorax and hemothorax may occur [12]; however, these are rare [3].

IMV patients with transudative PE who underwent drainage weaned from ventilation faster than those managed medically without TT [11]. However, no studies have compared hemodynamic/respiratory responses between IMV patients with exudative versus transudative PE.

This retrospective longitudinal study aimed to compare post-TT changes in respiratory and hemodynamic parameters in ICU patients on IMV, stratified by transudative versus exudative PE.

## 2. Materials and Methods

### 2.1. Study Design and Participants

This single-center, retrospective longitudinal study included adult men and women admitted to the ICU on IMV with PE undergoing TT between 1 January 2019, and 30 April 2024, at a high complexity center.

**Figure 1:**
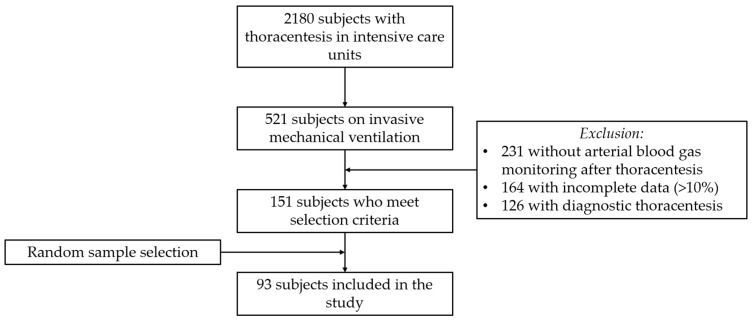
Flowchart of study participant selection.

Patients were identified via an institutional imaging-guided TT database. Those on IMV during the procedure were selected. Patients with incomplete data exceeding 10% in their medical records, a single indication for diagnostic thoracentesis, and pregnant patients were excluded (Figure 1).

Sample size was calculated using an expected mean ± SD of 144.2 ± 35.1 for PaO_2_/FiO_2_, reported in a recent study [6], with 5% precision (d), 95% confidence (α), target population of 151 TT patients, design effect of 1, and 10% non-response rate, yielding 93 subjects. Simple random sampling was used.

### 2.2. Data Collection

Demographic and clinical data were recorded, including age, sex, socioeconomic status, health insurance affiliation, medical history/comorbidities, ICU admission cause, days from admission to procedure, days on IMV prior to the procedure, need for hemodynamic support, laterality of PE, C-reactive protein, creatinine levels, ventilator mode, antibiotic therapy, and hospital length of stay. LVEF was obtained from the most recent echocardiogram performed during the ICU stay.

Thoracentesis was performed at the bedside in the intensive care unit using a sterile technique and real-time ultrasound guidance. A convex or micro-convex transducer was used to identify the pleural space, confirm fluid presence, and select the optimal puncture site while avoiding vascular structures and lung parenchyma. Local anesthesia was applied, and the procedure was performed using the Seldinger technique with a flexible pleural catheter for controlled drainage. The evacuated volume was monitored continuously, and drainage was interrupted in the event of hemodynamic instability or oxygen desaturation. All procedures were performed by intensive care physicians and/or radiologists with formal training in thoracic ultrasound. For this study, Light’s criteria, drained volume, procedure related complications, and need for additional PE management were documented. Follow-up measurements were taken at three time points: during the procedure, 12 h post-thoracentesis, and 24 h post-thoracentesis.

Each participant was assigned a unique identification code to ensure confidentiality. Data were consolidated into a master database containing all variables from the collection instrument. Concordance assessment and statistical validation of the database were performed. To ensure data accuracy, entries were double-checked, allowing rigorous refinement and organization of the final dataset.

### 2.3. Data Analysis

Categorical variables were analyzed using frequency distributions, absolute frequencies, and relative frequencies. Quantitative data were analyzed using numerical measures of central tendency and dispersion, with categorization and 95% confidence intervals (CI). Effect size between pre- and post-thoracentesis measurements was estimated using Cohen’s d for each clinical outcome parameter, with cut-off values interpreted as small (0.20), medium (0.50), and large (0.80) effects. For normally distributed parameters (Shapiro–Wilk test), Student’s t test (t statistic) was applied; for non-normally distributed parameters, Wilcoxon signed rank test (z statistic) was used, with effect size expressed via paired rank biserial correlation. Additionally, a stratified analysis was performed based on two drained volume groups (<500 mL and ≥500 mL), fluid type (Light’s criteria), and LVEF group. Statistical analysis was conducted using STATA 17.0^®^ (StataCorp, College Station, TX, USA).

## 3. Results

A total of 93 subjects meeting the inclusion criteria were enrolled. Most participants were over 60 years old, female sex, of middle socioeconomic status, and enrolled in the contributory health insurance system. Regarding medical history, 63.4% had hypertension, 34.4% diabetes mellitus, 32.3% heart failure, 30.1% chronic kidney disease, and 21.5% chronic obstructive pulmonary disease. Among the subjects with a history of chronic kidney disease, only three (10.7%) were receiving renal replacement therapy or hemodialysis. In the study population, 83.9% were admitted to the ICU and/or required invasive ventilatory support due to respiratory failure arising from medical causes (cardiovascular, oncologic, infectious, pulmonary, or other etiologies), whereas 16.1% were admitted to the ICU and/or required invasive ventilation due to surgical indications. The eight oncologic patients corresponded to six cases of tumor lysis syndrome and two cases of malignant hypercalcemia. In addition, in the clinical characteristics, hospital stays of ≥15 days and ≤7 days of ventilatory support until the time of the procedure were reported in greater proportion, 44.1% and 59.1%, respectively. Similarly, as a result of shock, 73.1% of patients required vasoactive support and 19.4% required inotropic support, 21.5% reporting LVEF ≤40% and 90.3% receiving antibiotic treatment (Table 1). PE was identified in 12.9% of the 93 subjects upon admission to the ICU.

Of the total study sample, 58.0% died during the hospital stay, and 1.1% were discharged with home mechanical ventilation. Regarding the characteristics of the thoracentesis, a right-sided or bilateral PE was most frequently observed, with transudate according to Light’s criteria and a complication-free procedure. Only 19.4% required a repeat thoracentesis, and 17.2% reported drainage exceeding 1000 mL (Table 2). Among the 18 patients who required repeated thoracentesis, a higher frequency of comorbidities and relevant clinical factors was observed compared with those who did not require a new drainage procedure. In this group, the most frequent antecedents were diabetes mellitus (38.9%), heart failure (33.3%), and liver disease (11.1%). In addition, 55.6% presented a cardiovascular reason for admission, 72.2% required vasopressor support, and most had a drained volume greater than 500 mL. Likewise, 66.7% of the pleural effusions corresponded to transudates.

In the population analyzed, 67 of the 93 patients achieved successful extubation during their subsequent clinical course following thoracentesis, resulting in an extubation rate of 72.0%.

Regarding the changes observed between the time of thoracentesis and the second post-procedure follow-up, statistically significant differences were reported in the parameters of FiO_2_ and PEEP; PaO_2_/FiO_2_ showed an average reduction of 4%, and PEEP decreased by 0.5 cmH_2_O, with low (0.43) and medium (0.75) effect sizes, respectively. PaO_2_/FiO_2_ increased by 27.1 points with a low effect size (0.39). Cardiovascular variables such as systolic, diastolic, and MAP showed an increase, while HR decreased, though without statistical significance (Table 3).

**Figure 2:**
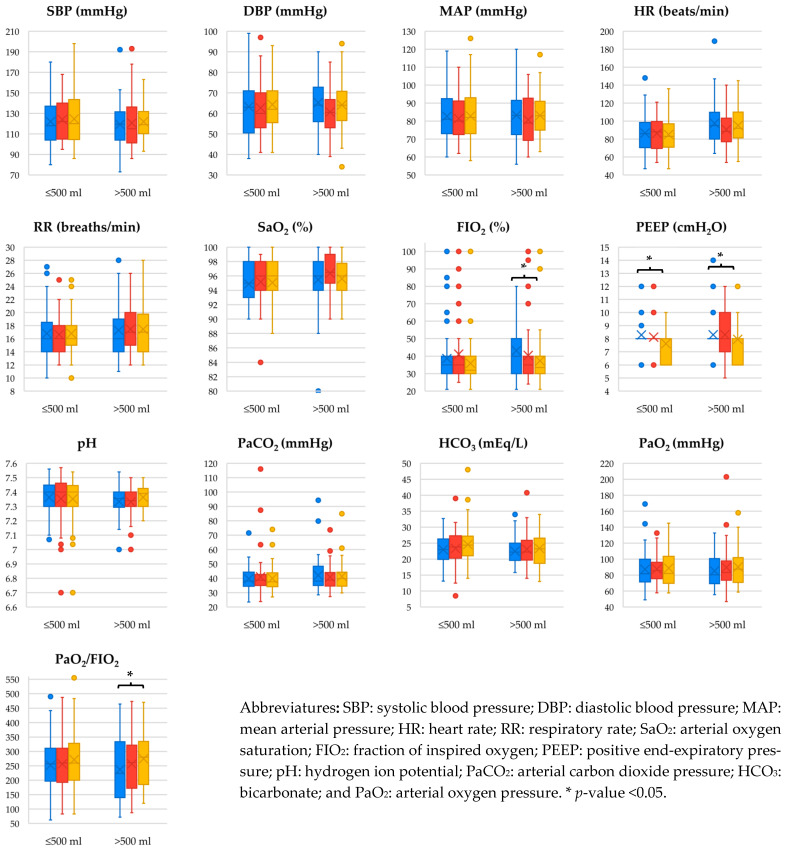
Changes in Cardiovascular and Respiratory Parameters After Thoracentesis According to Drained Volume. Measurement at the time of the procedure (blue), and follow-up at 12 h (red) and 24 h (yellow).

When stratifying the analysis of cardiovascular and respiratory parameters by drained volume, a similar trend was observed (Figure 2). FiO_2_ and PaO_2_/FiO_2_ showed statistically significant differences only in the >500 mL group, while PEEP showed differences in both groups. In the ≤500 mL group, only PEEP demonstrated a reduction, with a mean decrease of 0.4 cmH_2_O and a low effect size (0.45). In contrast, the >500 mL group showed an average FiO_2_ increase of 6% and PaO_2_/FiO_2_ increase of 38.0 points, with higher effect sizes (0.56 and 0.55, respectively). In this same group, PEEP decreased by 0.6 cmH_2_O with a medium effect size (0.57) after thoracentesis.

At the time of thoracentesis, the artificial airway in subjects receiving invasive mechanical ventilation most commonly consisted of an endotracheal tube (75 patients, 80.6%), whereas a smaller proportion had a tracheostomy cannula (18 patients, 19.4%). In these two groups, no significant differences were observed in complications, clinical outcomes, or length of hospital stay.

Similarly, subjects with LVEF ≤ 40% showed a statistically significant difference (*p* < 0.05) before and after thoracentesis in MAP (77.2 vs. 81.2 mmHg), albeit with a low effect size (0.31), and in PaO_2_ (81.5 vs. 115.4 mmHg), with a medium effect size (0.52). In the LVEF > 40% group, significant changes were reported in FiO_2_ (41.2% vs. 37.3%), PEEP (8.1 vs. 7.4 cmH_2_O), and PaO_2_/FiO_2_ (233.0 vs. 265.6), with effect sizes of 0.44 (low), 0.58 (medium), and 0.46 (low), respectively.

In the groups stratified by type of effusion according to Light’s criteria, subjects with exudative effusions showed no significant changes in parameter averages. Conversely, the transudate group exhibited a significant post-drainage impact (*p* < 0.05) in FiO_2_ (42.0% vs. 35.8%) with a medium effect size (0.59), PEEP (8.4 vs. 7.9 cmH2O) with a medium effect size (0.67), PaCO_2_ (42.4 vs. 39.8 mmHg) with a low effect size (0.27), and PaO_2_/FiO_2_ (242.7 vs. 274.6) with a low effect size (0.43).

## 4. Discussion

PE in IMV represents a significant clinical challenge due to its impact on respiratory mechanics, gas exchange, and in severe cases, hemodynamic stability. Its pathophysiology revolves around the inflammation–endothelium–capillary leak triad, with consequent activation of proinflammatory cytokines (IL-6, IL-8, and TNF-α) and mediators, such as angiopoietin 2, that increase vascular permeability, thereby altering Starling’s equilibrium and promoting fluid accumulation in the pleural space [13]. This mechanism elevates lung recoil pressures and causes refractory hypoxemia, which is associated with increased mortality and prolonged ICU stays [14,15]. Thoracentesis plays a key role in PE management, with significant diagnostic and therapeutic utility, although its ability to relieve dyspnea and characterize pleural fluid is well established [16]. A gap persists in documenting its effect on cardiopulmonary parameters in IMV patients, particularly its influence when stratified by drained volume and fluid type, as well as its impact on cardiac function, ventilator parameters, and oxygenation.

Evidence confirms that thoracentesis significantly improves oxygenation and ventilatory mechanics in critically ill patients. Studies such as that by Razazi et al. report an average increase of 59 points in PaO_2_/FiO_2_ within 24 h post-procedure [4], while Umbrello et al. demonstrated improvement in diaphragmatic function during ventilator weaning [17]. Similarly, Park et al. observed a benefit of 39 points in PaO_2_/FiO_2_ exclusively in surviving patients [15]. Our study results suggested the same trend, with an average increase of 27.1 points in PaO_2_/FiO_2_ (*p* < 0.01), 38.0 points in PE ≥ 500 mL, and significant reductions in ventilator requirements. This volume-dependent relationship aligns with the dose–response effect described by Razazi and Fang [4,18]. FiO_2_ showed an average reduction of 4% and PEEP decreased by 0.5 cmH_2_O. Our findings confirm a central physiological principle: evacuation of the pleural effusion releases compression on the lung parenchyma, improves the distensibility of the respiratory system, and reduces the pressure required to generate alveolar volume, which explains the clinical benefit of thoracentesis more robustly than any isolated average of ventilatory parameters. Consequently, the practical implication is not to apply a uniform numerical correction of PEEP after drainage, but to reinforce a management paradigm based on individualized titration, guided by the patient’s integrated response ventilatory mechanics, gas exchange, and hemodynamic stability rather than by the automatic application of a predetermined adjustment. Additionally, no significant changes were observed in blood pressure or HR, confirming immediate cardiovascular stability post-procedure.

Unlike previous studies focused on late outcomes such as mortality prognostic factors [14,15], our study centered on the immediate physiological response, demonstrating that thoracentesis generates early respiratory benefits within 24 h post-procedure, proportional to the drained volume, which could facilitate ventilator weaning. Our results, consistent with those of a recent systematic review in critically ill patients with PE, support the therapeutic role of thoracentesis by demonstrating its ability to simultaneously optimize ventilatory mechanics and gas exchange in the context of mechanical ventilation.

Our findings reveal a key aspect not previously documented in the medical literature: the physiological benefit of thoracentesis appears to depend on the nature of the PE. Patients with transudative effusions showed significant improvement in oxygenation and ventilatory mechanics, reflected in an increase in PaO_2_/FiO_2_ (from 242.7 to 274.6), as well as reductions in FiO_2_, PEEP, and PaCO_2_ (*p* < 0.05). This observation suggests that when PE is transudative, respiratory dysfunction predominantly results from mechanical compression on relatively compliant lung parenchyma. Fluid evacuation consequently improves lung aeration, optimizing ventilation/perfusion (V/Q) matching by reducing intrapulmonary shunt secondary to compressive atelectasis [15]. In contrast, the absence of statistically significant improvement in the exudative effusion group suggests that in these cases, pleural fluid results from a local inflammatory process (pleural or parenchymal) that represents the primary limiting factor for lung re-expansion and gas exchange improvement [19,20]. Our findings indicate that while thoracentesis remains necessary for exudative effusions, its immediate physiological impact is less pronounced compared to transudative effusions.

Regarding hemodynamic analysis, while it has been postulated that progressive PE accumulation compromises hemodynamics by reversing the physiologically negative intrapleural pressure—potentially leading to adverse effects such as increased intrathoracic pressure, reduced venous return, and elevated pulmonary vascular resistance [10]—the use of sedatives in mechanically ventilated patients additionally negatively impacts hemodynamic variables in critically ill patients, often requiring increased vasopressor support [21].

The reported requirement for vasopressor support in patients undergoing thoracentesis is relatively low, with rates ranging from 9% to 15% [18,22,23], while other authors report no need for vasopressors [17]. In our cohort, this percentage was considerably higher (73.1%), suggesting our population had more severe hemodynamic compromise at the time of the procedure. Nevertheless, the intervention proved safe, as no statistically significant changes were observed in hemodynamic variables (systolic, diastolic, and MAP), as well as HR post-thoracentesis. This stability finding is not isolated and aligns with observations by Ahmed et al., who similarly reported no significant variations in these parameters in invasively monitored ventilated patients after thoracentesis [24].

Another noteworthy finding emerged in subgroup analysis: a striking physiological dichotomy dependent on cardiac function. In patients with systolic dysfunction (LVEF ≤ 40%), thoracentesis was not only safe but also therapeutic, inducing statistically significant increases in MAP (from 77.2 to 81.2 mmHg) and PaO_2_ (from 81.5 to 115.4 mmHg). We propose this beneficial effect may be mediated by reduced left ventricular afterload. In contrast, patients with preserved systolic function (LVEF > 40%) showed no significant hemodynamic changes, though they did exhibit respiratory benefits. Thus, we suggest this procedure may optimize hemodynamics in mechanically ventilated patients with left ventricular dysfunction.

Finally, thoracentesis is widely recognized as safe in critically ill patients [25,26]. Recent evidence reports pneumothorax incidence of 0.8% and hemothorax of 0.6% in this intervention, attributed to standardized real-time ultrasound guidance and smaller caliber catheters [6]. Consistent with this trend, our study reported low adverse event rates. Observed pneumothorax (<4%) and hemothorax (<2%) rates confirm the procedure’s safety even in high-complexity populations like ours.

The interpretation of our findings should consider current evidence regarding indications and safety of thoracentesis in critically ill patients. Recent updates highlight that decision-making for pleural drainage must be individualized and based on clinical context rather than a fixed volumetric threshold: primary considerations include respiratory compromise, uncertain effusion etiology, or imaging (ultrasound/CT) evidence of significant fluid accumulation [27,28]. Therapeutic thoracentesis should be balanced against potential risks, such as pneumothorax, bleeding or re-expansion pulmonary edema (RPE); importantly, use of real-time ultrasound guidance has been repeatedly shown to increase procedural success and significantly reduce complication rates; this represents the current standard of care in pleural interventions [29,30]. Although older practices advocated limits (e.g., 1–1.5 L per session), these are not strongly supported by high-quality evidence; contemporary series describe safe drainage of larger volumes under close monitoring, especially when guided by ultrasound and performed by experienced operators [29,31].

Moreover, for effusions with likely transudative origin (e.g., heart failure and hypoalbuminemia), conservative medical management remains an appropriate first-line strategy; invasive drainage should be reserved for patients with persistent symptoms, ventilatory or hemodynamic impairment, or failure of medical therapy [28,32]. In this context, our observation of a volume-dependent benefit (≥500 mL), especially in transudative effusions, aligns with the physiologic expectation that effective reduction of intrathoracic pressure and lung re-expansion may improve oxygenation and hemodynamics. Nevertheless, given the heterogeneity of the critically ill population and the potential for rare but serious complications, our findings support a selective, individualized approach integrating clinical severity, fluid volume, ultrasound guidance, and operator expertise, rather than adoption of a universal drainage threshold.

This study has important methodological strengths, particularly its exclusive focus on mechanically ventilated patients an understudied subgroup and comprehensive evaluation of pleural fluid type, LVEF value, oxygenation parameters, ventilatory mechanics, and hemodynamic profile. However, this study has limitations inherent to its retrospective, longitudinal, descriptive, and single-center design. The absence of a comparative group precludes the establishment of causal relationships or the evaluation of the impact of thoracentesis on major clinical outcomes such as the duration of mechanical ventilation, length of hospital stay, or mortality. The results should be interpreted as descriptive of changes in clinical and ventilatory parameters. These findings highlight the need for multicenter prospective studies to more rigorously evaluate the impact of thoracentesis on clinically relevant outcomes.

## 5. Conclusions

In critically ill mechanically ventilated patients, TT proved to be a safe and effective intervention for improving ventilatory and oxygenation parameters, particularly in transudative pleural effusions where improvement was significant. Results showed a volume-dependent effect, with greater benefits observed in those with drained volumes ≥500 mL. Additionally, the subgroup with LVEF ≤ 40% showed significant improvement in MAP and PaO_2_ compared to those with LVEF > 40%. The observed hemodynamic stability, even in patients with cardiovascular instability or requiring vasoactive support, reinforces the procedure’s safety profile in complex clinical scenarios.

## Figures and Tables

**Table 1:** Sociodemographic and clinical characteristics.

Characteristic	Category	N (93)	%
Age	≤60 years	25	26.9%
	>60 years	68	73.1%
	Mean (±SD)	65.4 (±17.6)	
Sex	Female	48	51.6%
	Male	45	48.4%
Socioeconomic status	Low (1–2)	22	23.7%
	Medium (3–4)	61	65.6%
	High (5–6)	10	10.8%
Health insurance	Subsidized	18	19.4%
	Contributory	74	79.6%
	Special	1	1.1%
Medical history	Hypertension (HTN)	59	63.4%
	COPD	20	21.5%
	Asthma	1	1.1%
	Diabetes mellitus	32	34.4%
	Chronic kidney disease	28	30.1%
	Obesity	23	24.7%
	Heart failure	30	32.3%
	Liver disease	6	6.5%
	Cerebrovascular disease	6	6.5%
	Atrial fibrillation	15	16.1%
	Other comorbidities	44	47.3%
Reason for ICU admission	Surgical	15	16.1%
	Cardiovascular	33	35.5%
	Infectious	30	32.3%
	Oncologic	8	8.6%
	Other	7	7.5%
Days from admission to procedure	≤7 days	35	37.6%
	8–14 days	17	18.3%
	≥15 days	41	44.1%
	Mean (±SD)	14.3 (±11.3)	
Days on mechanical ventilation	≤7 days	55	59.1%
	8–14 days	20	21.5%
	≥15 days	18	19.4%
	Mean (±SD)	7.8 (±7.5)	
Hemodynamic support	Vasopressors	68	73.1%
	Inotropes	18	19.4%
Left Ventricular Ejection Fraction (LVEF)	≤40%	20	21.5%
	>40%	51	54.8%
	Not reported	22	23.7%
	Mean (±SD)	49.6 (±13.4)	
C-reactive protein (CRP)	1.0–10.0 mg/dL	5	5.4%
	10.01–50.0 mg/dL	15	16.1%
	>50.0 mg/dL	59	63.4%
	Not reported	14	15.1%
	Mean (±SD)	116.2 (±108.0)	
Creatinine	≤0.4 mg/dL	6	6.5%
	0.5–1.3 mg/dL	53	57.0%
	≥1.4 mg/dL	34	36.6%
	Mean (±SD)	1.6 (±2.1)	
Ventilatory mode	Volume-controlled (VC)	24	25.8%
	Pressure-controlled (PC)	58	62.4%
	CPAP + PS	8	8.6%
	VC + Plus	1	1.1%
	Other	2	2.2%
Antibiotic therapy	No	9	9.7%
	Yes	84	90.3%
Hospital length of stay	≤7 days	5	5.4%
	8–14 days	13	14.0%
	≥15 days	75	80.6%
	Mean (±SD)	36.9 (±27.1)	

Abbreviatures: HTN, Hypertension; COPD, Chronic Obstructive Pulmonary Disease; LVEF, Left Ventricular Ejection Fraction; VC, Volume-controlled; PC, Pressure-controlled CPAP + PS; Continuous Positive Airway Pressure + Pressure Support; and ±SD, Standard Deviation.

**Table 2:** Characteristics Related to Thoracentesis.

Characteristic	Category	N	%
Light’s Criteria	Exudate	20	21.5%
	Transudate	50	53.8%
	Not evaluated	23	24.7%
Pleural effusion laterality	Right	34	36.6%
	Left	25	26.9%
	Bilateral	34	36.6%
Drained volume	Not quantified	13	14.0%
	0–500 mL	40	43.0%
	501–1000 mL	25	26.9%
	1001–2000 mL	15	17.2%
Procedure-related complications	Pneumothorax	3	3.2%
	Hemothorax	1	1.1%
	Hypotension	1	1.1%
	Shock	4	4.3%
	No complications	84	90.3%
Additional management required for pleural effusion	No intervention	59	63.4%
	Chest tube placement	4	4.3%
	Thoracoscopy	7	7.5%
	Thoracotomy	2	2.2%
	Pleurodesis	3	3.2%
	Repeat thoracentesis	18	19.4%

**Table 3:** Changes in Cardiovascular and Respiratory Parameters After Thoracentesis.

Variable	Pre-Intervention					Post-Intervention 1					Post-Intervention 2					Δ (*Diff.*)	Test Statistic (t or z)	Effect Size *	*p*-Value
	$\bar{X}$	SD	Med	IQR		$\bar{X}$	SD	Med	IQR		$\bar{X}$	SD	Med	IQR					
Systolic BP (SBP), mmHg	120.9	21.4	119.0	104.0	−133.0	122.6	22.4	120.0	104.0	−140.0	123.5	22.3	120.0	108.5	−140.0	2.6	−934.0	0.10	0.353
Diastolic BP (DBP), mmHg	64.1	12.7	63.0	54.0	−72.0	61.8	11.8	60.0	53.0	−70.0	64.2	12.5	63.0	56.0	−71.0	0.1	−0.054	0.01	0.957
Mean BP (MAP), mmHg	82.9	13.7	83.0	73.0	−91.0	81.1	12.7	79.0	71.0	−91.7	83.0	13.4	82.0	74.5	−91.5	0.1	−0.007	0.07	0.995
Heart rate (bpm)	91.3	23.0	90.0	77.5	−103.0	88.2	19.1	88.0	74.0	−100.5	89.4	20.8	87.0	74.0	−101.5	−1.9	2109.5	0.08	0.530
Respiratory rate (rpm)	17.0	3.7	16.0	14.0	−19.0	17.0	3.1	16.0	14.5	−18.0	17.1	3.4	16.0	14.5	−19.0	−0.1	1117.5	0.08	0.591
SpO_2_ (%)	95.1	4.2	96.0	93.5	−98.0	95.7	3.9	96.0	94.5	−98.0	95.3	3.8	96.0	94.0	−98.0	0.2	−0.443	0.05	0.659
FiO_2_ (%)	40.6	15.5	35.0	30.0	−50.0	40.8	17.9	35.0	30.0	−40.0	36.5	13.9	32.0	30.0	−40.0	−4.1	1679.5	0.43	0.002
PEEP (cmH_2_O)	8.3	1.6	8.0	8.0	−8.0	8.2	2.0	8.0	8.0	−8.0	7.8	2.4	8.0	6.0	−8.0	−0.5	406.0	0.75	0.001
pH	7.4	0.1	7.4	7.3	−7.4	7.3	0.1	7.4	7.3	−7.4	7.4	0.1	7.4	7.3	−7.4	0.0	1128.5	0.14	0.299
PaCO_2_ (mmHg)	40.7	10.8	38.0	34.7	−44.5	41.0	12.4	38.7	35.0	−43.9	40.5	9.3	38.5	34.5	−44.0	0.2	2114.0	0.01	0.924
HCO_3_^−^ (mEq/L)	22.7	4.5	22.5	19.7	−26.0	24.8	23.8	83.7	74.9	−96.7	26.1	13.3	24.0	20.7	−26.8	3.4	1495.0	0.29	0.991
PaO_2_ (mmHg)	86.7	23.9	81.0	71.0	−99.9	87.2	12.6	23.3	19.8	−26.9	95.7	49.3	85.0	70.6	−101.9	9.0	1893.0	0.16	0.126
PaO_2_/FiO_2_	245.8	103.2	249.0	156.0	−316.3	256.6	94.3	260.0	183.0	−314.0	272.9	101.1	264.0	197.6	−329.5	27.1	−2.742	0.39	0.001

* For the Student’s *t*-test (t-statistic), effect size is expressed as Cohen’s d. For the Wilcoxon test (z-statistic), effect size is expressed as the matched-pairs rank-biserial correlation. Legend: $\bar{X}$, mean; Med, median; SD: standard deviation; IQR: interquartile range; SpO_2_: arterial oxygen saturation; FiO_2_: fraction of inspired oxygen; PEEP: Positive End-Expiratory Pressure; pH: hydrogen potential; PaCO_2_: arterial carbon dioxide pressure; HCO_3_^−^: bicarbonate; and PaO_2_: arterial oxygen pressure.

## Data Availability

The original contributions presented in the study are included in the article, and further inquiries can be directed to the corresponding author.

## Abbreviations

The following abbreviations are used in this manuscript:

TT	Therapeutic Thoracentesis
ICU	Intensive Care Unit
PE	Pleural Effusion
IMV	Invasive Mechanical Ventilation
